# Genomic and Phenotypic Characterization of *Streptomyces sirii* sp. nov., Amicetin-Producing Actinobacteria Isolated from Bamboo Rhizospheric Soil

**DOI:** 10.3390/microorganisms12122628

**Published:** 2024-12-19

**Authors:** Yuliya V. Zakalyukina, Vera A. Alferova, Arina A. Nikandrova, Albina R. Kiriy, Alisa P. Chernyshova, Marsel R. Kabilov, Olga A. Baturina, Mikhail V. Biryukov, Petr V. Sergiev, Dmitrii A. Lukianov

**Affiliations:** 1 Department of Soil Science, Lomonosov Moscow State University, 119991 Moscow, Russia; 2 Belozersky Institute of Physico-Chemical Biology, Lomonosov Moscow State University, 119991 Moscow, Russia; alferovava@gmail.com (V.A.A.); petya@genebee.msu.ru (P.V.S.); 3 Shemyakin-Ovchinnikov Institute of Bioorganic Chemistry of the Russian Academy of Science, 117997 Moscow, Russia; abeidy@mail.ru; 4 Center for Molecular and Cellular Biology, 121205 Moscow, Skolkovo, Russia; arinanikandrova@mail.ru (A.A.N.); lukianovda@my.msu.ru (D.A.L.); 5 Department of Biology, Lomonosov Moscow State University, 119991 Moscow, Russia; metrim@gmail.com; 6 Institute of Biomedical Problems of the Russian Academy of Sciences, 123007 Moscow, Russia; albinabelik32@gmail.com; 7 Department of Chemistry, Lomonosov Moscow State University, 119991 Moscow, Russia; 8 Institute of Chemical Biology and Fundamental Medicine Siberian Branch of the Russian Academy of Sciences, 630090 Novosibirsk, Russia; kabilov@niboch.nsc.ru (M.R.K.); baturina@niboch.nsc.ru (O.A.B.); 9 Scientific Center for Translational Medicine, Sirius University of Science and Technology, 354340 Sochi, Russia

**Keywords:** amicetin, BGCs, polyphasic taxonomy, rhizosphere soil, *Streptomyces*

## Abstract

In our large-scale search for antimicrobial-producing bacteria, we isolated an actinomycete strain from rhizospheric soil of *Bambusa vulgaris*. The strain designated BP-8 showed noticeable antibacterial activity. BP-8 was subjected to a whole-genome analysis via a polyphasic taxonomy approach, and its antibacterial metabolite was identified by HRLS-MS. The results of the physiological and morphological analyses indicated that BP-8 is an aerobic, neutrophilic, mesophilic organism that is tolerant to 8% NaCl and can use a wide range of carbohydrates. It forms curly sporophores with a warty surface. The results of the phylogenetic and average nucleotide identity analyses and in silico DNA–DNA hybridization calculation indicated that BP-8 represents the type strain of a novel *Streptomyces* species. A comparative in silico analysis of the genome sequences of BP-8 and its closest related strains revealed the presence of genes encoding chemotaxonomic markers characteristic of *Streptomyces*. The antibacterial compound was identified as amicetin. Genomic mining also revealed more than 10 biosynthetic gene clusters that have not been described previously and may lead to the discovery of new valuable compounds. On the basis of these results, strain BP-8^T^ (=VKM Ac-3066^T^ = CCTCC AA 2024094^T^) is proposed as the type strain of the novel species *Streptomyces sirii* sp. nov.

## 1. Introduction

In light of the increasing threat posed by pathogenic bacteria that exhibit multidrug resistance, scientists are facing the challenge of finding new strategies and techniques to develop effective antibiotics [1].

Mycelial actinomycetes, in particular those belonging to the genus *Streptomyces*, are undeniably the leaders among prokaryotic microorganisms in terms of the variety of natural substances they produce. These products have antimicrobial, antifungal, antitumor, anti-inflammatory, and insecticidal activities, accounting for two-thirds of all antibiotics currently on the market [2]. Despite the fact that streptomycetes have captivated the attention of scientists since the 1940s, the full extent of their biosynthetic capabilities remains largely undiscovered [3]. Genome analyses of *Streptomyces* strains revealed that each strain has a large and linear chromosome encoding more than 20 secondary metabolite biosynthetic gene clusters (BGCs), even if they are known to produce only a few known compounds. It is believed that many of these BGCs encode new natural products, and further study of *Streptomyces* may lead to the discovery of compounds with unique chemical structures and novel modes of action [4].

The genus *Streptomyces* is the largest taxon within the phylum Actinomycetota (class Actinomycetes, order Kitasatosporales, family Streptomycetaceae), including 730 validly published and correctly described species (https://lpsn.dsmz.de/genus/streptomyces (accessed on 21 September 2024)). Members of the genus *Streptomyces* are aerobic, Gram-stain-positive, multicellular bacteria that form extensively branched substrate mycelia and develop aerial hyphae with chains of spores in the mature stage [5].

Soils are the traditional source of streptomycete isolation. However, recently, the number of new *Streptomyces* species has been continuously increasing due to the discovery of new strains in extreme habitats, such as marine sediments, caves, deserts [6], mountains, and ice-covered areas [7]. Researchers also focus on the symbiotic relationships between streptomycetes and invertebrates [8,9], based on the hypothesis that *Streptomyces* have evolved to produce antimicrobial compounds that are not harmful to their eukaryotic hosts [10,11].

Streptomyces can act as beneficial symbionts for plants [12], called, in this case, plant growth promoting streptomycetes (PGPS) [13], controlling phytopathogens [14] and synthesizing other biologically active compounds, such as extracellular hydrolytic enzymes, siderophores, and phytohormones. There are many examples in the literature showing how streptomyces stimulate plant growth, increase plant resistance to stress, and improve plant health by enriching rhizospheric soils [15].

In the course of our large-scale search for cultivated producers of antimicrobial substances, we isolated an actinomycete strain from the rhizosphere of *Bambuza vulgaris*. The strain was subjected to a whole-genome analysis, and we were also able to identify an antibacterial metabolite produced by this strain. Here, we analyze the results of a polyphasic taxonomic study on this strain, designated BP-8^T^, and propose that this strain represents a novel species of the genus *Streptomyces*.

## 2. Materials and Methods

### 2.1. Collection, Isolation, and Preservation

Soil samples from the rhizosphere of bamboo, about 5 cm below the surface, were collected in Sirius, Russia (43.415912 N, 39.925498 W), in July 2021. ISP 3 agar medium [16], supplemented with nystatin and nalidixic acid at final concentrations of 250 μg/mL and 10 μg/mL, was used to isolate the bacteria from the phylum Actinomycetota. The plates were incubated for 2–3 weeks at 28 °C until colonies with different morphological characteristics appeared. The colonies with grey aerial mycelium that demonstrated antagonistic activity against Gram-positive bacteria were generalized as the BP-8 morphotype, which was selected for further study. The long-term storage of the strain was carried out via a spore suspension in glycerol (20%, *v*/*v*) at −80 °C.

### 2.2. Phenotypic Characterization

Cultural characteristics of BP-8 were determined after incubation for 14 days at 28 °C on yeast extract–malt extract agar (ISP 2), oatmeal agar (ISP 3), inorganic salt–starch agar (ISP 4), glycerol–asparagine agar (ISP 5), peptone–yeast extract iron agar (ISP 6), and tyrosine agar (ISP 7) [16].

Spore chain morphology was examined by light microscopy (Fisherbrand AX-502, Thermo Fisher Scientific) at 100–1000× magnification. For scanning electron microscopy, we used oatmeal agar plugs with 12-day BP-8, fixed with 2.5% glutaraldehyde, dehydrated using ethanol series, and subsequently critical-point-dried. The samples were coated with gold–palladium before observation with a JSM-6380LA (JEOL, Tokyo, Japan).

Growth at different temperatures (8, 15, 20, 28, 37, 45, and 50 °C) and at different NaCl concentrations (1%, 5%, and 8%) were carried out, and the tolerable pH range from 4.5 to 9.5 was determined on Organic broth 79 after 2 weeks of incubation, as described earlier [17].

Carbon source utilization was examined on minimal medium ISP9 [16] supplied with bromocresol purple and 1% of either arabinose, fructose, galactose, glucose, lactose, maltose, mannitol, mannose, myo-inositol, raffinose, rhamnose, sorbitol, sucrose, trehalose, or xylose.

### 2.3. Genome Features and Phylogenomic Analysis

Genomic DNA was extracted from pure culture using the ZymoBIOMICS DNA Microprep Kit (Zymo Research, Irvine, CA, USA). To obtain long reads, DNA was sheared using a g-tube (Covaris, Woburn, MA, USA) with a medium size of fragment distribution of about 8 kb. MinION sequencing of fragmented DNA was conducted according to the 1D Native bar-coding genomic DNA protocol with EXP-NBD103, SQK-LSK108, and R9 flowcell kits (Oxford Nanopore Technologies, Oxford, UK) in the Genomics Core Facility (ICBFM SB RAS, Novosibirsk, Russia). The raw long reads were base-called and demultiplexed by Guppy v4.2.3 (https://community.nanoporetech.com/downloads (accessed on 1 May 2024)). Adaper trimming was performed using Porechop_ABI v0.5.0 [18] and Snikt v0.5.0 [19], and quality filtration was carried out by Fitlong v0.2.1 (https://github.com/rrwick/Filtlong (accessed on 1 May 2024)).

In the case of short reads, DNA was fragmented to an average size of 600 bp in a microTUBE AFA fiber snap-cap tube in S2 instrument (Covaris, Woburn, MA, USA). The paired-end (PE) library was constructed using dual-index NEBNext multiplex oligonucleotides and a NEBNext Ultra II DNA library prepkit for Illumina (New England BioLabs, Ipswich, MA, USA). The DNA library was sequenced with a reagent kit v3 (600 cycle) on a MiSeq sequencer (Illumina) in the Genomics Core Facility (ICBFM SB RAS, Novosibirsk, Russia). The reads were quality trimmed (QV > 20), and adapter sequences were removed by Cutadapt v3.5 [20].

The whole genome of strain BP-8 was parallel assembled from long reads using Flye v2.9.3 [21], Miniasm v0.3 [22] with Minipolish v0.3 [23], and Raven v1.8.3 [24]. The consensus assembly from multiple input assemblies was produced by Trycycler v0.5.4 [25]. For genome polishing by filtered long and short reads, Medaka v1.11.3 (https://github.com/nanoporetech/medaka (accessed on 1 May 2024)) and Polypolish v0.6.0 [26] with Polca v4.1.1 [27] were used, respectively. The quality of the assembled genome was checked by BUSCO v5.8.0 with the dataset bacte-ria_odb10 [28] and CheckM v1.2.3 [29].

The automatic functional annotation results were obtained using the NCBI Prokaryotic Genome Annotation Pipeline [30]. The de novo workflow from GTDB-tk v2.4.0 [31] with reference genomes (GTDB r220) [32] were used for taxonomic classifications of the genome. ANI (average nucleotide identity) and AF (alignment fraction) values were also calculated in GTDB-tk.

The assembly is available in GenBank with its accession number. The full-length 16S rRNA gene sequences of strain BP-8 (1529 bp) were submitted to the GenBank database (as. no. OR636680.1).

The genome sequence data were uploaded to the Type (Strain) Genome Server (TYGS), a free bioinformatics platform available under https://tygs.dsmz.de (accessed on 21 September 2024), for a whole genome-based taxonomic analysis [33]. Information on nomenclature and synonymy was provided by TYGS’s sister database, the List of Prokaryotic names with Standing in Nomenclature (LPSN, available at https://lpsn.dsmz.de (accessed on 21 September 2024)) [34]. 

For the in silico digital DNA, DNA hybridization (DDH) values were calculated by using the GGDC method, with the recommended formula 2, available at the TYGS web service (https://tygs.dsmz.de/ (accessed on 21 September 2024)).

An average nucleotide identity analysis with a sequence pairing based on blast+ (ANIb) was carried out using the JSpeciesWS server: https://jspecies.ribohost.com/jspeciesws (accessed on 21 September 2024) [35]. Those scores were calculated to compare BP-8 with its closest type strains with complete genome.

### 2.4. In Silico Chemotaxonomy

Genes encoding key enzymes and proteins involved in the synthesis of fatty acids, polar lipids, and isoprenoid quinones were identified in the BP-8^T^ genome and compared to sequences of orthologous genes from related strains of *Streptomyces* deposited in GenBank.

### 2.5. Analysis of Bioactive Compound Biosynthetic Gene Clusters

Secondary metabolite biosynthetic gene clusters in the complete genome of strain BP-8 were identified with the bacterial version of antiSMASH 7.0 (https://antismash.secondarymetabolites.org/ (accessed on 21 September 2024)).

### 2.6. Antibacterial Activity Testing on Reporter Strains

To obtain a sufficient amount of the active compound for detailed bioactivity studies, strain BP-8^T^ was cultured in four 750 mL Erlenmeyer flasks with 250 mL of liquid Organic medium 79 at 28 °C with shaking (250 rpm) for 5 days. Culture liquids were separated from biomass by centrifugation at 20,000× *g* for 20 min (Centrifuge 5810 R, Rotor FA-45-6-30, Eppendorf, Hamburg, Germany), and the supernatants were subjected to solid-phase extraction and primary fractionation on LPS-500-H sorbent (LLC “Technosorbent”, Moscow, Russia) using water–acetonitrile mixtures as eluents.

Antibacterial activity was assessed using the reporter strain *E. coli* JW5503 ΔtolC pDualRep2, as previously described [36]. The overnight culture of the reporter strain was diluted with fresh LB medium to an optical density of 600 nm (OD600) of 0.05–0.1. The culture was transferred to LB agar plates that had 100 mg/mL ampicillin applied. On an agar plate with the lawn of one colony of the reporter strain BP-8, culture broth was applied along with two control antibiotics: erythromycin (Ery, 2 μg) and levofloxacin (Lev, 0.05 μg). The macrolide antibiotic erythromycin disrupts protein biosynthesis in the bacterial cell (expression of the Katushka2S protein is visualized during scanning), and levofloxacin is a DNA gyrase inhibitor that induces the cell’s SOS response (expression of the TurboRFP protein is visualized during scanning). The plates were incubated at 37 °C overnight and then scanned by ChemiDoc scanner (Bio-Rad, Hercules, CA, USA) in the modes ‘Cy3-blot’ for RFP and ‘Cy5-blot’ for Katushka2S. When scanning, the Katushka2S protein’s signal was displayed in red, and the RFP protein’s signal was displayed in green.

### 2.7. Purification and Identification of Amicetin

The obtained BP-8^T^ culture broth was subjected to gravity-force solid-phase extraction on an LPS-500-H sorbent (polyvinilbenzen, pore size 50–1000 Å) with elution by 0, 10, 20, 25, 30, 35, 40, and 50% aqueous acetonitrile solutions. The pDualrep2 reporter system was used to analyze the activity in the fractions. The fraction eluted by 25% acetonitrile demonstrated the highest inhibitory activity and induced Katushka2S expression, indicating the presence of a protein synthesis inhibitor. This fraction was subjected to further purification via HPLC (Interchim Puriflash 4250, isocratic elution 25% of MeCN 0.1% TFA for 8 min, then isocratic elution 55% of MeCN 0.1% TFA for 6 min) using a ZORBAX SB-C18 (Agilent Technologies, Santa Clara, CA, USA) column (7 μm, 21.2 × 250 mm). The collected fractions were analyzed by a pDualrep2 reporter system, and an individual peak (retention time 12 min) with antibacterial activity was collected.

HPLC analysis and fractionation were performed with the Vanquish Flex UHPLC System using a Diode Array Detector (Thermo Fisher Scientific, Waltham, MA, USA), equipped with a Luna^®^ 5 μm C18(2) 100 Å, 250 × 4.6 mm column (Phenomenex, Torrance, CA, USA). Mass spectra were collected using a maXis II 4G ETD mass spectrometer (Bruker Daltonics, Bremen, Germany) and UltiMate 3000 chromatograph (Thermo Fisher Scientific, Waltham, MA, USA), equipped with an Acclaim RSLC 120 C18 2.2 μm 2.1 × 100 mm column (Thermo Fisher Scientific, Waltham, MA, USA). The spectrum registration mode and ESI ionization mode were determined with a full scan from 100 to 1500 *m*/*z*, and MS/MS was performed with the selection of the three most intense ions, with the dissociation type CID 10–40 eV and nitrogen collision gas.

## 3. Results

The search for actinomycete strains with the ability to inhibit protein synthesis was conducted in the Federal Territory Sirius (Krasnodarskiy kray, Russia) in various locations, where samples of seawater, sandy sediments from beaches, and soils under various plants were collected.

Among others, strains were detected, isolated both from beach sand and from soils, characterized by16S rRNA and phenotypic similarities, and as it was later identified, synthesizing the antibacterial antibiotic amicetin. We chose one isolate BP-8, originating from bamboo rhizospheric soil, for whole-genome sequencing and the subsequent bioinformatic analysis.

### 3.1. Classification of Strain BP-8^T^

#### 3.1.1. Phylogeny

Initially, the approximate taxonomic classification of the BP-8 strain was determined based on an analysis of the 16S rRNA gene sequence (1529 bp) by comparison to sequences of type strains in the GenBank database (on 12 September 2024). It showed high similarity *to Streptomyces libani* subsp. *libani* DSM 40555^T^ (99.48%), *Streptomyces auratus* DSM 41897^T^ (99.35%), *Streptomyces inhibens* DSM 106197^T^ (99.35%), and *Streptomyces lydicus* ATCC 25470^T^ (99.12%). The 16S rRNA-based phylogenies inferred from GBDP distances using FastME 2.1.6.1 are shown in Figure S1.

**Figure 1:**
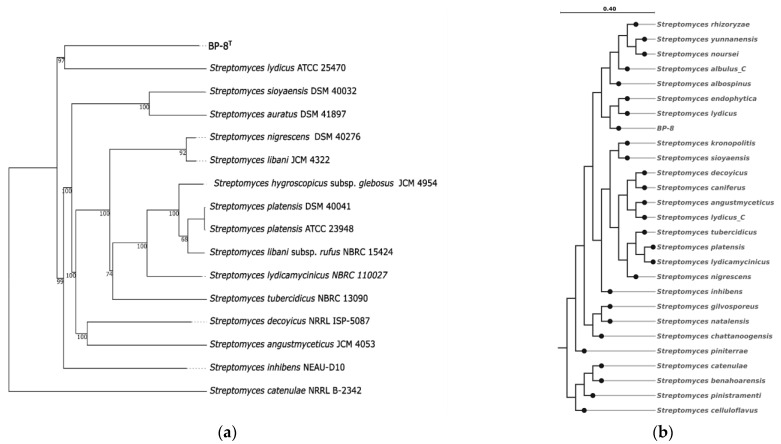
Phylogenetic trees of *Streptomyces* sp. BP-8^T^ and type strains of the related species: (**a**) tree inferred with FastME 2.1.6.1 [5] from GBDP distances calculated from genome sequences. The branch lengths are scaled in terms of GBDP distance formula d5. The numbers above branches are GBDP pseudo-bootstrap support values > 60% from 100 replications, with an average branch support of 91.5%. The tree is rooted at the midpoint. (**b**) Part of phylogenomic tree for *Streptomyces* genera constructed with de novo workflow of GTDB-tk.

Widely used polyphaser studies largely take the similarity of the 16S rRNA gene as a basis, but despite its usefulness for solving taxonomic issues in the past, this gene contains only a limited number of features and, thus, like any other single gene, can produce trees with many statistically unconfirmed branches. Progress in sequencing technologies makes it possible to use genome-wide sequences to construct phylogenetic trees, which provides much greater reliability of identification. The GTDB genome tree inferred from an aligned concatenated set of 120 single-copy marker proteins for *Streptomyces* genera, as well as a taxonomic tree from the TYGS server using the Genome BLAST Distance Phylogeny (GBDP) method, agree with each other in supporting a clade formed of BP-8^T^ and *Streptomyces lydicus* (Figure 1a,b).

#### 3.1.2. Phenotypic Properties

**Figure 2:**
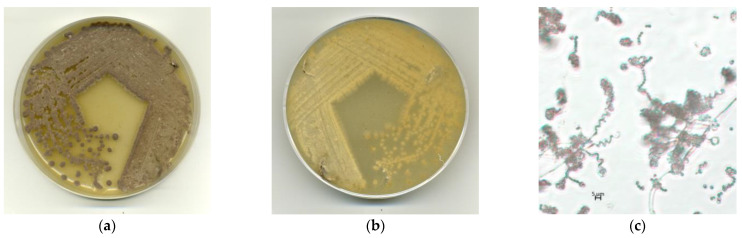
Cultural and morphological properties of BP-8^T^ on ISP3 media after 10 days at 28 °C: (**a**) color of aerial mycelium, (**b**) color of substrate mycelium, (**c**) spiral chains of spores (×1000 magnification).

Good growth was observed on all the tested media. The color of the aerial mycelium varied from white or light grey to grey, and that of the substrate mycelium varied from colorless to pale yellowish (Figure 2a,b). No diffusible pigments or melanin were observed on any of the tested media. Strain BP-8^T^ formed spiral chains of spores after a 10-day incubation (Figure 2c).

**Figure 3:**
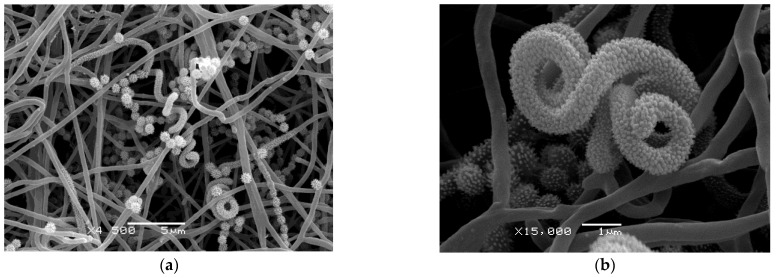
Scanning electron micrographs of strain BP-8^T^ on ISP3 agar for 12 days at 28 °C: (**a**) spiral chains of spores; (**b**) warty surface of non-mature spore chain among smooth aerial hyphae.

The spore surface of BP-8^T^ on ISP3 agar was examined using scanning electron microscopy. The smooth surface of the aerial mycelium became warty due to special surface proteins in those places where spore-bearing hyphae are formed (Figure 3a). They curl into spirals, in which individual globular spores (approximately 0.67–0.90 µm in diameter) form and separate over time (Figure 3b).

The tolerable pH range of BP-8^T^ was from 4.5 to 9.5 and with an optimum near-neutral point. The BP-8^T^ did not grow on arabinose and rhamnose. It grew well on fructose, galactose, glucose, lactose, maltose, mannitol, mannose, myo-inositol, raffinose, sorbitol, sucrose, and trehalose but only slowly on xylose. Salt tolerance of BP-8^T^ was found up to 8% NaCl. The strain grew at temperatures between 20 and 37 °C, with the best temperature range being between 28 and 2 °C.

The strain was sensitive to amikacin (30 µg), gentamicin (10 µg), tobramycin (10 µg), vancomycin (30 µg), and erythromycin (15 µg), including trimethoprim–sulfamethoxazole (25 µg) and clindamycin (2 µg), which showed a bacteriostatic effect; it was resistant to betalactams such as amoxicillin (30 µg), aztreonam (30 µg), piperacillin–tazobactam (110 µg), cefoxitin (30 µg), ceftazidime (30 µg), and cefepime (30 µg) (Figure S2).

#### 3.1.3. Genomic Characterization

The genome was checked by Busco (C:99.2%[S:96.8%,D:2.4%],F:0.8%,M:0.0%,n:124) and CheckM (completeness 100%, contamination 1.25%), which showed high assembly quality.

The size of the genomic DNA of BP-8^T^ was 8,633,236 bp. This was in accordance with the genome sizes of its closest relatives (7,046,907–9,534,571 bp) (Table 1). The 71.5 moL% G+C content of BP-8^T^ was consistent with the average G+C content of species of the genus *Streptomyces* (66–78%) [37].

The ANIb scores ranged from 80.59 to 87.29% (Table 1). The most closely related type-strain genomes were those of *Streptomyces inhibens* NEAU-D10^T^ (87.29%), *S. lydicus* ATCC 25470^T^ (86.50%), and *Streptomyces libani* subsp. *libani* JCM 4322 (86.38%). This was clearly below the species-delineating consensus threshold (95–96%) [38].

Digital DDH indicated that the DNA–DNA relatedness between *Streptomyces* sp. BP-8^T^ and the fifteen type strains is 25.5–35% (Table 1), which is much lower than the cut-off point of 70% recommended for the assignment of bacteria strains to the same genomic species [33].

Therefore, the ANI and dDDH values supported the conclusion that strain BP-8^T^ represented a distinct species in the genus *Streptomyces*.

The results of the GTDB-tk analysis led to the same conclusion. The most closely related species to BP-8^T^ by AI and AF (alignment fraction) are *Streptomyces inhibens* NEAU-D10^T^ and *Streptomyces lydicus* NRRL ISP-5461. These two species have an ANI of 88.68 and 88.56 and an AF of 0.55 and 0.54, respectively. The GTDB uses an ANI greater than 95% and an AF greater than 0.65 to classify bacteria into specific species.

#### 3.1.4. In Silico Chemotaxonomy

Fatty acids, polar lipids, and isoprenoid quinones are common subjects for chemotaxonomic analyses of actinomycetes. We performed in silico chemotaxonomy among *Streptomyces* species based on a comparative genomic approach as an alternative to the more traditional chemotaxonomy [39].

*Streptomyces* utilizes a type II FAS to catalyze the de novo synthesis of fatty acids. The majority are made from branched starters such as isobutyryl, isovaleryl, and anteisovaleryl units to give odd- and even-numbered fatty acids with a methyl branch at the ω-terminus (80–90% of total fatty acid content); the rest are synthesized from unbranched starters such as acetyl and butyryl units [40]. An extended analysis of all the annotated streptomycetes genomes revealed that some of the genes encoding the core enzymes involved in saturated fatty acid biosynthesis lie in a conserved fatty acid biosynthesis (fab) cluster that comprises the following genes: fabD, fabH, acpP, and fabF. This cluster has been characterized and its relationship with the fatty acid biosynthetic pathway confirmed through genetic and biochemical experiments. Further, the alignment of several actinomycete genomic regions containing this universal set of genes was determined, indicating a high degree of conservation in the evolution of this essential biosynthetic pathway [40]. Indeed, all the genes of this operon are present in the genomes of BP-8^T^ and its close type strains (Table S1), which indicates the common pathways of fatty acid synthesis characteristic of the *Streptomyces* genus.

ACP S-malonyltransferase (FabD) catalyzes the transfer of malonate to acyl-carrier- protein (ACP) and then converts the acyl groups into thioester forms, which are characteristic of acyl intermediates in fatty acid synthesis and are strictly required for the condensation reactions catalyzed by β-ketoacyl-ACP synthetase. The enzyme 3-ketoacyl-ACP synthase III (FabH), a key enzyme for bacteria growth, catalyzes the last step of the initiation of bacterial fatty acid synthesis. The similarity of orthologous genes coding FabH is sufficiently high and accounts for 94–95% (Table S1), while *fab*D of BP-8^T^ is most closely related to the gene in *Streptomyces sioyaensis* DSM 40032^T^ (89.35%), as well as *S. lydicus* ATCC 25470^T^, *S. libani* subsp. *libani* ATCC 23732^T^, and *S. auratus* DSM 41897^T^ (≈88%).

The presence of diaminopimelate epimerase (dapF), responsible for the interconversion of the LL- and meso-isomers of DAP, was also annotated in genomes of BP-8^T^ and related *Streptomyces* type strains (Table S1), whose cell wall is characterized by containing large amounts of LL-DAP [5]. The amino acid sequences of the *mur*E genes, encoding the UDP-N-acetylmuramoylalanyl-D-glutamate-2,6-diaminopimelate ligase in BP-8^T^ and related strains, are also characterized by a high level of similarity (Table S1).

Since the predominant glycerophospholipids in *Streptomyces inhibens* [41], *Streptomyces lydicamycinicus* NBRC 110027^T^ [42], and *Streptomyces staurosporininus* [43] are DPG, PE, OH-PE, and PI (Table S2), we analyzed the genome of the strain BP-8^T^ for the presence of genes encoding these types of phospholipids. Phosphatidylserine descarboxylase (encoded by psd), required for the synthesis of phosphatidylethanolamine (PE), was annotated in all the considered genomes (Table S3). CDP-diacylglycerol—inositol 3-phosphatidyltransferase [EC 2.7.8.11] needed for the synthesis of PI (phosphatidylinositol) and cardiolipin synthase [EC 2.7.8.41], required in the synthesis of cardiolipin (DPG), respectively, were present in all the genomes including BP-8^T^ (Table S3). 

Menaquinones are one of the major isoprenoid quinones required in bacterial electron transport systems. The length and degree of saturation in isoprenoid chains of menaquinones are considered to be key taxonomic markers in the classification of Actinobacteria. The enzyme, encoded by the menaquinone reductase gene (menJ), confers the saturation of menaquinones: it catalyzes the reduction of a single double bond in the isoprenoid tail of menaquinone (MK-9), likely the beta-isoprene unit, forming the predominant form of menaquinone, MK-9(II-H2). The deletion of the menJ gene leads to the production of MK-9 instead of MK-9(H2) [44].

The gene menJ was misidentified by GenBank as encoding the ageranylgeranyl reductase family protein, as noted earlier by Montero-Calasanz and colleagues [39], but accessing the Unipro database (P9WNY8) made it possible to verify the amino acid sequence as menaquinone-9-β-reductase [EC 1.3.99.38] in all the considered genomes (Table S1).

### 3.2. Biosynthetic Gene Clusters Coding for Secondary Metabolites and Antibiotic Resistance Genes

The genome of *Streptomyces* sp. BP-8^T^ contains seven type I polyketide synthases (PKSs), two type II PKSs, one type III PKS, four NRPSs, one trans-AT PKS, and one heterocyst glycolipid synthase-like PKS gene cluster, as listed in Table S4.

**Figure 4:**
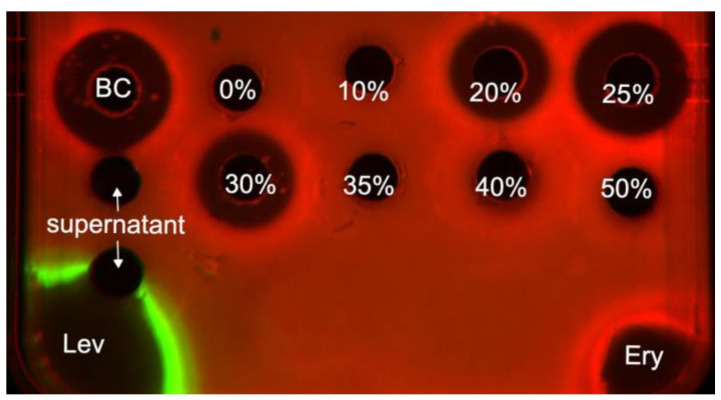
Induction of a two-color reporter system sensitive to inhibitors of ribosome progression and DNA replication. Erythromycin (Ery), levofloxacin (Lev), and the compounds obtained during the broth culture purification via solid-phase extraction were placed on an agar plate with *E. coli* ΔtolC cells transformed with a pDualrep2 plasmid. The fluorescence of the *E. coli* lawn was scanned at 553/574 nm for RFP fluorescence and 588/633 nm for Katushka2S. The induction of Katushka2S expression is triggered by translation inhibitors, and RFP is regulated by the SOS response to DNA damage. BC—broth culture of BP-8^T^; 0, 10, 20, 25, 30, 35, 40, and 50%—elution with acetonitrile solutions in water of indicated volume concentrations.

We detected a BGC, exhibiting high similarity with amicetin BGC (BGC0000953) (Figure 4), as a product of NPKS. T2PKS were identified as BGCs for spore pigment and α-naphtocyclinoic acid, while iso-migrastatin with congeners were identified as products of trans-acyltransferase polyketide synthase. The products of other clusters are still unclear due to the lack of data in the MiBIG and similar databases (Table S4).

The genome of the strain BP-8^T^ was found to contain the genes responsible for both specific and nonspecific mechanisms of bacterial resistance to antibacterial drugs (Table S5). The presence of the gene encoding the metallo-beta-lactamase family protein is an example of a specific mechanism of protection against beta-lactams, which was confirmed by the results of testing for antibiotic sensitivity (see Section 3.1.2). One of the common mechanisms of bacterial drug resistance is the export of drug compounds by efflux pumps. The BP-8^T^ strain was found to possess two genes of ABS efflux pumps (Table S5).

### 3.3. Antimicrobial Activity of Streptomyces sirii BP-8^T^

The antimicrobial activity of the BP-8^T^ broth culture was studied on an *E. coli* ΔtolC pDualrep2 (AmpR) reporter strain. In addition, a similar agar plate test was repeated after each step of isolation and purification of the active compound. All BP-8^T^ samples exhibiting antibacterial activity (inhibition zone diameter > 8 mm) demonstrated strong induction of the reporter protein Katushka2S (Figure 4), similar to that observed with erythromycin, indicating that the antibacterial compound in BP-8^T^ may negatively affect protein synthesis in bacterial cells.

### 3.4. Identification of Antibacterial Compound Produced by Strain BP-8^T^

**Figure 5:**
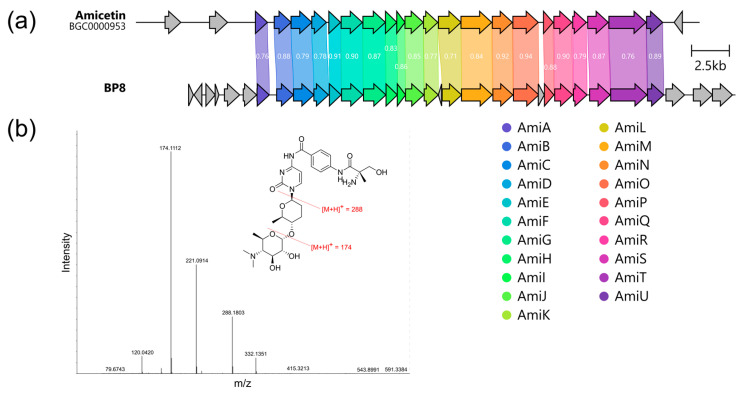
Analysis of bioactive metabolites from the strain BP-8^T^: (**a**) a comparison of amicetin BGC (BGC0000953) with region 7 in the BP-8^T^ complete genome sequence, generated using clinker tool [46]. Homologous genes are highlighted with colors, and labels indicate identity of the genes. (**b**) MS2 fragmentation spectrum of [M+H]^+^ ion (*m*/*z* 619.3078) of the isolated compound; structure of amicetin and characteristic [45] fragmentation patterns.

Taking into account the plausible inhibition of protein synthesis by the active component, as well as the presence of genes demonstrating high similarity to amicetin BGC (BGC0000953), detected by AntiSMASH in the whole genome of BP-8^T^ (Figure 5a), we identified the active compound by primary fractionation of the supernatant on the LPS-500-H sorbent and HR-LCMS.

As expected, the analysis of the active fraction using HR-LCMS revealed an ion with m/z 619.3078 (calculated for amicetin C_29_H_42_N_6_O_9_ [M+H]^+^ 619.3092 Da, Δ2.3 ppm). Fragmentation of this ion (Figure 5b) contained fragment ions with m/z 288 and 174, characteristic for amicetin [45].

## 4. Discussion

Several innovative strategies can be employed to enhance the efficiency and expedite the screening of known compounds. One approach involves screening non-cultivated strains, although obtaining sufficient antibiotics for detection presents significant challenges [47]. An additional method entails the identification of novel antibiotic producers and cultivating these strains alongside target organisms [48]. Genome mining represents another valuable option for compound discovery [49,50]. Furthermore, the application of deep learning techniques offers an alternative pathway for improving screening processes [51]. Utilizing reporter systems is another strategy that can facilitate the identification of antibiotic mechanisms of action [36]. Reporter systems also serve to accelerate the dereplication process, enabling the determination of mechanisms of action that may have previously been mischaracterized. For instance, this approach proved beneficial in our recent studies involving the translation inhibitor tetracenomycin X [52], whose mechanism of action had been inaccurately described [53,54].

Guided by this approach, we studied cultured actinomycetes from various natural habitats and discovered the strain BR-8 from the rhizosphere of bamboo, which showed antibacterial activity through a disruption of protein synthesis (Figure 4).

The new isolate has been identified as a member of the genus *Streptomyces* based on an analysis of its16S rRNA sequence (Figure S1). Although it showed high similarity to *Streptomyces libani* subsp. *libani* DSM 40555^T^ (99.48%), the 16S rDNA sequence similarity of >99.0% does not necessarily guarantee that both strains belong to the same species [42]. An analysis of the assembled genome by two independent approaches (GBDP by DNA–DNA relatedness and average nucleotide identity for related strains of Streptomyces with >99% similarity in 16S rDNA sequences, as well as GTDB by analysis of 120 genes taking into account ANI and alignment fraction) confirmed that the studied BP-8T should be a new species in *Streptomyces* (Figure 1). Based on these findings, we propose *Streptomyces sirii* sp. nov. for BP-8^T^. A description of the new species is given below.

Modern taxonomy, based on the analysis of complete genomes, has a high level of resolution for identifying bacterial species. This eliminates the need for time-consuming methods such as in vitro DNA–DNA hybridization and the analysis of biochemical markers using “wet chemistry” methods. Traditional chemotaxonomy can only confirm that the properties of a new member of a genus are similar to those of other members of that genus, information that can be predicted based on its phylogenetic placement within the genus [55].

Currently, researchers increasingly use chemotaxonomy in silico, which allows for both faster taxon description and more accurate and consistent taxonomic reports [39]. This simple and fast approach, which is sufficient to confirm the affiliation of new isolates with well-studied taxonomic groups such as the genus *Streptomyces*, allowed us to complement the phylogenomic results with an analysis of genes encoding the proteins (Table S1) responsible for the synthesis and metabolism of major chemotaxonomic markers (Table S2).

It is important to mention that for many type strains of *Streptomyces*, which were initially described prior to the 1970s, their chemotaxonomic characteristics have not been established. For the type strains of *Streptomyces lydicus* De Boer et al. 1956, *Streptomyces nigrescens* (Sveshnikova 1957) Pridham et al. 1958, and *Streptomyces libani* subsp. *libani* Baldacci and Grein 1966, there are no data of chemotaxonomic tests in the primary sources. The strain *Streptomyces decoyicus* NRRL ISP-5087^T^ was firstly described as *Streptomyces hygroscopicus* var. *decoyicus* in 1959 [56] without conducting chemotaxonomic studies, and was later reclassified in 2010, however, also without chemotaxonomical analyses [57].

By the bioactivity-guided isolation methods used here, we found that BP-8^T^ could produce amicetin (Figure 5 and Figure S3), belonging to a group of disaccharide nucleoside antibiotics and active against a number of both Gram-negative and Gram-positive bacteria, particularly *Mycobacterium tuberculosis*, and also against herpesvirus I type and poliovirus [58]. Despite the fact that amicetin was discovered more than 70 years ago [59], its ability to selectively bind to the P-site of the bacterial ribosome and inhibit bacterial translation makes it a promising candidate for the development of synthetic modifications and new anti-tuberculosis drugs [60].

It is worth noting that BP-8^T^ is very rich in genes for polyketide synthases, non-ribosomal peptide synthases, and other proteins that are responsible for the production of secondary metabolites (Table S4). The fact that the exact nature of the final products produced by these BGCs has not yet been identified makes a more detailed study of *Streptomyces sirii* BP-8^T^ a promising avenue for discovering new, biotechnologically valuable molecules.

## 5. Conclusions

Thanks to a comprehensive analysis of the genome of the actinomycete strain BP-8^T^ isolated from rhizospheric soil, we were able to establish that it represents a new species of *Streptomyces* according to the accepted criteria for differentiation. A chemotaxonomic analysis in silico also confirmed its similarity with the closest representatives of the *Streptomyces* genus.

Genomic mining revealed a wide variety of genetic clusters for secondary metabolites, many of which had not been previously described. The identification of the amicetin gene cluster was confirmed by detecting this translation inhibitor among the metabolites produced by this strain during laboratory cultivation. We also discovered genes responsible for the strain’s resistance to common clinical antibiotics.

The described properties can serve as a starting point for further research in order to explore the biotechnological potential of BP-8^T^ based on the genomic data that have already been collected but have not yet been thoroughly analyzed.

## 6. Description of *Streptomyces sirii* sp. nov.

*Streptomyces sirii* sp. nov. (si’ri.i. L. gen. n. *sirii*, referring to Sirius University where the soil sample was collected).

Aerobic, Gram-staining-positive, mesophilic, filamentous actinobacterium that forms curly sporophores with warty surface on the aerial hyphae. When maturing, the spore chains break up into globular spores of 0.67–0.90 µm in diameter. Grows well on ISP2-ISP6 forming from white to grey aerial mycelium; no pigment production was detected on the media examined.

Grows at 20–37 °C (optimally at 28 °C), pH 4.5–8.5 (optimally at pH 7.0), and in the presence of 0–8% (*w*/*v*) NaCl.

Acid production was observed on ISP9 agar plates with the following carbon sources: glucose, galactose, fructose, myo-inositol, lactose, maltose, mannitol, mannose, raffinose, sucrose, sorbitol, and trehalose.

Susceptible to amikacin (30 µg), clindamycin (2 µg), gentamycin (10 µg), erythromycin (15 µg), tobramycin (10 µg), and vancomycin (30 µg).

The genome of *Streptomyces sirii* contains genes responsible for the synthesis of fatty acids, phospholipids, menaquinones, and peptidoglycan, characteristic of representatives of the genus *Streptomyces*. The in silico analysis revealed high similarity between orthologous genes involved in the biosynthesis of chemotaxonomically significant biopolymers.

The type strain is BP-8^T^ (=VKM Ac-3066^T^ = CCTCC AA 2024094^T^), which was isolated from bamboo rhizospheric soil collected from Imeretinsky Bay (Sirius, Russia).

The DNA G+C content of the type strain is 71.5%, and the genome size is 8,633,236 bp. The GenBank accession number for the draft genome sequence is CP147982.1. The accession number of the 16S sequence is OR636680.1.

## Figures and Tables

**Table 1:** The characteristics and a comparison of the whole genome of BP-8^T^ with those of its closest type strains.

Strain	dDDH with Strain BP-8^T^ (%)	ANI with Strain BP-8^T^ (%)	Size (bp)	DNA G+C Content (mol%)	Accession No.
BP-8^T^	--	--	8,633,236	71.5	CP147982.1
*Streptomyces inhibens* NEAU-D10	35	87.29	9,462,258	70.5	GCA_003389455
*Streptomyces decoyicus* NRRL ISP-5087	34	86.27	8,398,847	70.7	GCA_000715775
*Streptomyces lydicus* ATCC 25470	33.8	86.50	7,935,716	70.2	GCA_004125245
*Streptomyces nigrescens* DSM 40276	33.2	86.36	9,534,571	70.8	GCA_027626975
*Streptomyces libani* subsp. *libani* JCM 4322	33.2	86.38	9,115,867	71.0	GCA_014649275
*Streptomyces sioyaensis* DSM 40032	32.7	85.82	7,842,713	71.6	GCA_004122735
*Streptomyces lydicamycinicus* NBRC 110027	32.6	85.89	8,313,448	71.2	GCA_000829715
*Streptomyces hygroscopicus* subsp. *glebosus* JCM 4954	32.6	85.92	8,379,736	71.1	GCA_014656235
*Streptomyces auratus * DSM 41897	32.6	85.81	8,438,454	71.2	SAMN05877744
*Streptomyces tubercidicus* NBRC 13090	32.6	85.59	7,928,609	70.7	GCA_009811635
*Streptomyces platensis* ATCC 23948	32.5	85.83	8,501,012	71.1	GCA_008704855
*Streptomyces libani* subsp. *rufus* NBRC 15424	32.5	85.79	8,624,950	71.1	GCA_009811615
*Streptomyces angustmyceticus* JCM 4053	32.4	85.61	8,116,382	72.2	GCA_019933235
*Streptomyces platensis* DSM 40041	32.3	85.76	8,400,476	71.2	GCA_002119195
*Streptomyces catenulae* NRRL B-2342	25.5	80.59	7,046,907	73.0	GCF_000718015

## Data Availability

The GenBank accession numbers for the 16S rRNA gene sequence and the genome assembly of strain BP-8^T^ are OR636680.1 and CP147982.1, respectively.

## Supplementary Materials

The following supporting information can be downloaded at https://www.mdpi.com/article/10.3390/microorganisms12122628/s1, Figure S1: Neighbor-joining phylogenetic tree of strain BP-8^T^ and related *Streptomyces* species; Figure S2: Antibiotic susceptibility testing of strain BP-8^T^; Table S1: Genes encoding the main chemotaxonomic markers of *Streptomyces* sp. BP-8^T^ and related species; Table S2: Fatty acids, isoprenoid quinones, and polar lipid profiles of strain BP-8^T^-related type streptomycete strains; Table S3: Amicetin clusters of *Streptomyces vinaceusdrappus* NRRL 2363 and *Streptomyces* sp. BP-8^T^; Table S4: Polyketide synthases (PKSs) and non-ribosomal peptide synthetases (NRPSs) in the gene clusters of *Streptomyces* sp. BP-8^T^; Table S5: Antibiotic resistance genes in the genome of *Streptomyces* sp. BP-8^T^; Figure S3: HPLC of the active fraction.
