# Supplementary material for: Genomic and Phenotypic Characterization of Streptomyces sirii sp. nov., Amicetin-Producing Actinobacteria Isolated from Bamboo Rhizospheric Soil
Source: Microorganisms. 2024 Dec 19;12(12):2628. doi: 10.3390/microorganisms12122628 (PMC11677201; doi:10.3390/microorganisms12122628)
Supplement: Supplementary file 1 [file microorganisms-12-02628-s001.zip › microorganisms-3340938-supplementary.pdf]

Genomic and Phenotypic Characterization of *Streptomyces sirii* sp.nov., Amicetin-Producing Actinobacteria Isolated from Bamboo Rhizospheric Soil

Yuliya V. Zakalyukina [1,*], Vera A. Alferova [2,3], Arina A. Nikandrova [4,5], Albina R. Kiriy [6], Alisa P. Chernyshova [3,7], Marsel R. Kabilov [8], Olga A. Baturina [8], Mikhail V. Biryukov [5,9], Petr V. Sergiev [2,4,7] and Dmitrii A. Lukianov [4,7]

Supplementary Materials

Content

Figure S1. Neighbor-joining phylogenetic tree of strain BP-8^T and related *Streptomyces* species 2

Figure S2. Antibiotic susceptibility testing of strain BP-8^T 3

Table S1. Genes encoding the main chemotaxonomic markers of *Streptomyces* sp. BP-8^T and related species 4

Table S2. Fatty acids, isoprenoid quinones, and polar lipid profiles of strain BP-8^T-related type streptomycete strains 5

Table S3. Amicetin clusters of *Streptomyces vinaceusdrappus* NRRL 2363 and *Streptomyces* sp. BP-8^T 6

Table S4. Polyketide synthases (PKSs) and nonribosomal peptide synthetases (NRPSs) in the gene clusters of *Streptomyces* sp. BP-8^T 7

Table S5. Antibiotic resistance genes in the genome of Streptomyces sp. BP-8^T 8

Figure S3. HPLC of the active fraction 8

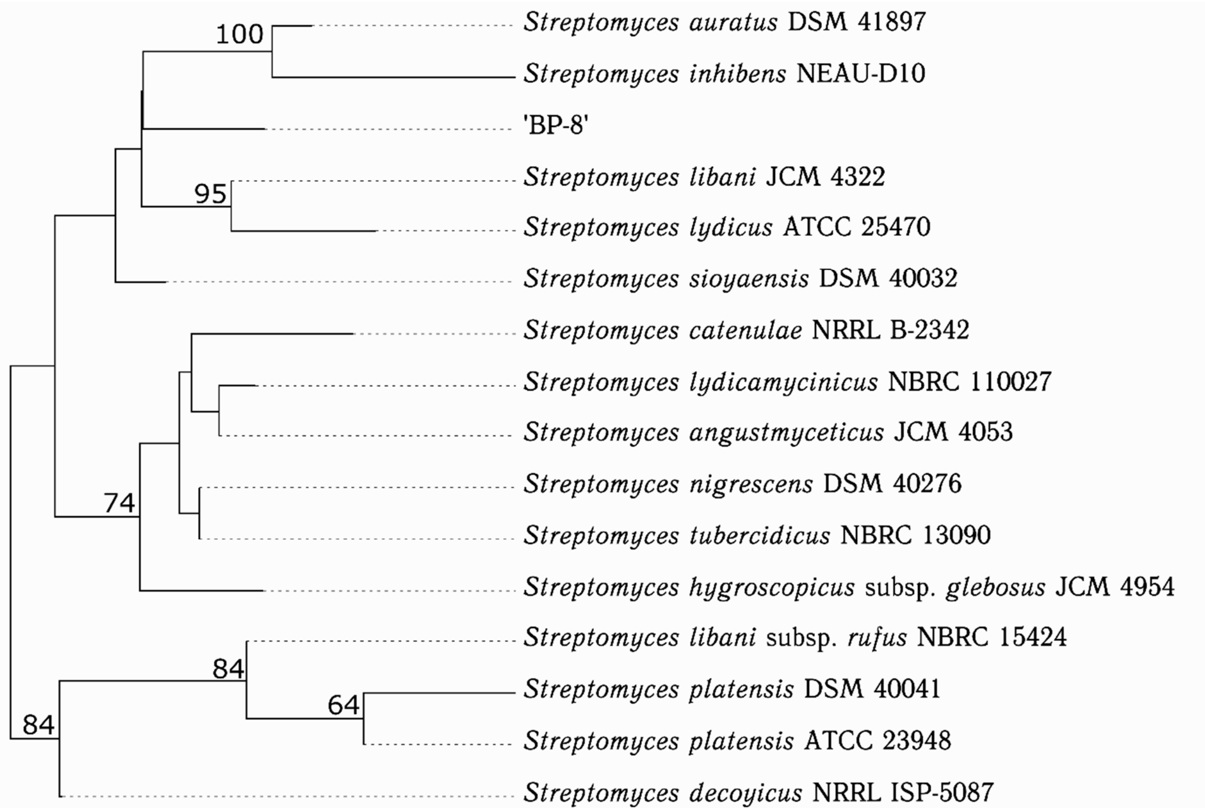

Figure S1. Tree inferred with FastME 2.1.6.1 from GBDP distances calculated from 16S rDNA gene sequences. The branch lengths are scaled in terms of GBDP distance formula d5. The numbers above branches are GBDP pseudo-bootstrap support values > 60 % from 100 replications, with an average branch support of 60.7 %. The tree was rooted at the midpoint.

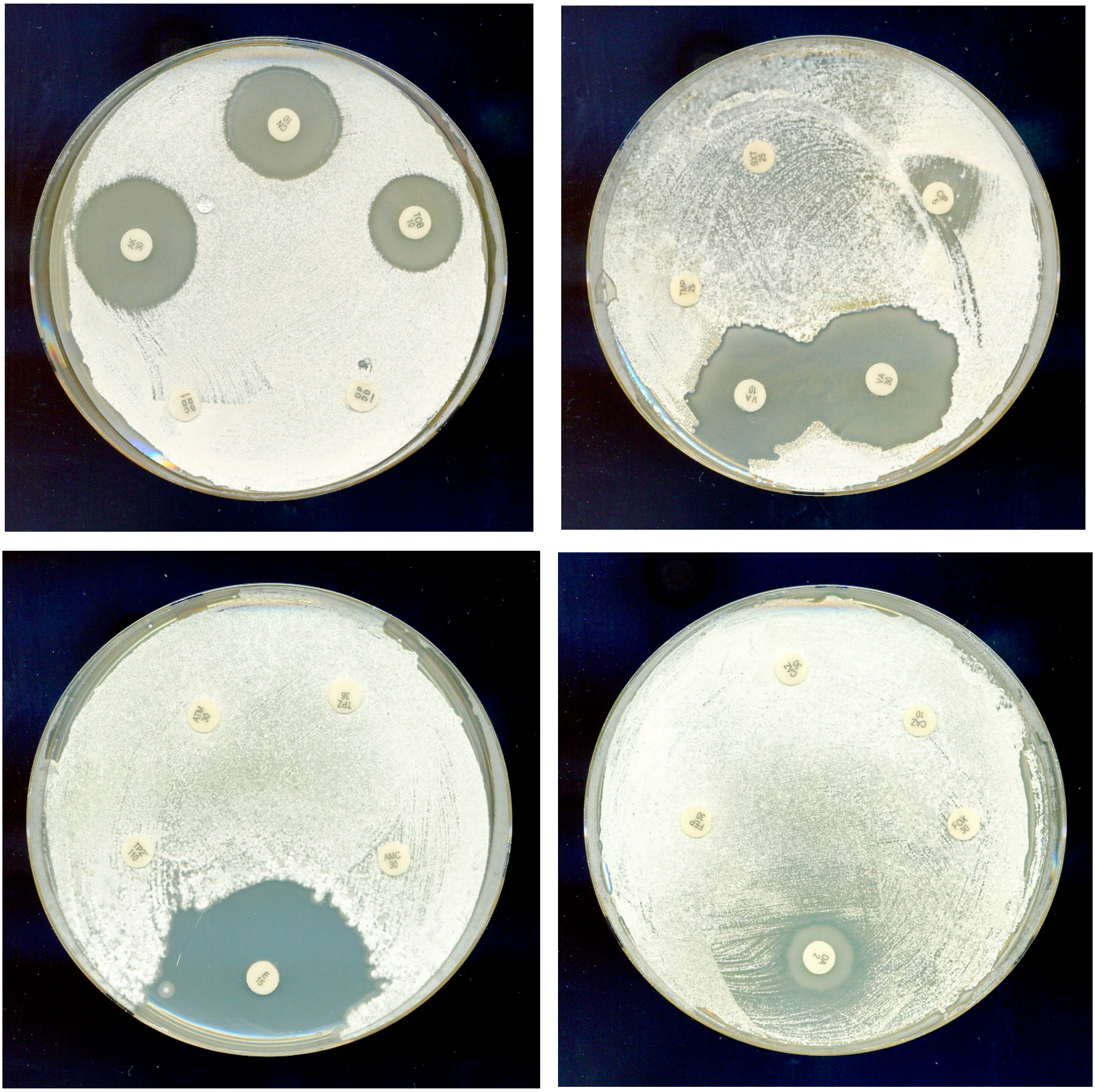

Figure S2. Antibiotic susceptibility testing of strain BP-8^{T}: AK 30, amikacin 30 μg; CN 10, gentamicin 10 μg; TOB 10, tobramycin 10 μg; VA 30, vancomycin 30 μg; E 15 erythromycin 15 μg; SXT 25, trimethoprim/sulfamethoxazole 25 μg; DA 2, clindamycin 2 μg; AMC 30, amoxicillin 30 μg; ATM 30, aztreonam 30 μg; TPZ 110, piperacillin/tazobactam 110 μg; FOX 30, cefoxitin 30 μg; CAZ 30, ceftazidime 30 μg; FEP 30, cefepime 30 μg.

Table S1. Genes encoding the main chemotaxonomic markers of *Streptomyces* sp. BP-8^T and related species

Gene	Accession GenBank	Length, aa	Protein	EC number	Similarity of orthologous genes in related type strains, %			
					Streptomyces decoyicus NRRL 2666^T	*Streptomyces inhibens* NEAU-D10^T	*Streptomyces lydicamycinicus* NBRC 110027^T	*Streptomyces sioyaensis* DSM 40032^T
Fatty acid biosynthesis								
fabD	WXK75861.1	777	ACP S-malonyltransferase	2.3.1.39	37.36%	39.52%	41.10%	89.35%
fabH	WXK76924.1	348	Beta-ketoacyl-ACP synthase III	2.3.1.180	95.98%	95.11%	95.11%	94.83%
acpP	WXK76925.1	83	Acyl carrier protein		53.62%	97.56%	97.56%	--
fabF	WXK76926.1	428	Beta-ketoacyl-ACP synthase II	2.3.1.179	92.15%	94.63%	--	84.11%
Glycerophospholipid metabolism								
psd	WXK80424.1	215	Phosphatidylserine decarboxylase	4.1.1.65	99.53%	99.53%	99.07%	99.53%
pgsA	WXK75667.1	202	CDP-diacylglycerol-phosphatidylglycerol phosphatidyltransferase	2.7.8.5	91.64%	93.10%	91.13%	95.05%
CDIPT	WXK76015.1	218	CDP-diacylglycerol-inositol 3-phosphatidyltransferase	2.7.8.11	96.79%	97.25%	96.33%	95.43%
plsC	WXK76052.1	222	1-acyl-sn-glycerol-3-phosphate acyltransferase	2.3.1.51	96.40%	96.40%	96.85%	95.05%
CrCLS1	WXK81246.1	131	Cardiolipin synthase	2.7.8.41	77.86%	74.05%	75.00%	80.15%
Peptidoglycan biosynthesis								
murE	WXK76612.1	586	UDP-N-acetylmuramoylalanyl-D-glutamate-2, 6-diaminopimelate ligase	6.3.2.13	93.12%	87.56%	91.54%	93.35%
dapF	WXK79879.1	291	Diaminopimelate epimerase	5.1.1.7	93.61%	92.58%	90.72%	90.72%
Menaquinone biosynthesis								
menB	WXK77759.1	253	1,4-dihydroxy-2-naphthoyl-CoA synthase	4.1.3.36	87.95%	90.36%	86.35%	86.75%
menG	WXK77925.1	232	Demethylmenaquinone methyltransferase	2.1.1.163	97.41%	96.12%	96.98%	96.12%
menJ	WXK77922.1	435	Menaquinone-9-β-reductase	1.3.99.38	96.11%	95.83%	95.16%	94.13%

Table S2. Fatty acids, isoprenoid quinones, and polar lipid profiles of strain BP-8^T-related type streptomycete strains

	Streptomyces inhibens NEAU-D10	*Streptomyces lydicamycinicus* NBRC 110027^T	*Streptomyces staurosporininus* NRRL B-24850^T
Predominant FA			
Iso-$C_{16:0}$	21.0 %	predominant	19.5 %
$C_{16:0}$	17.8 %	predominant	17.0 %
anteiso-$C_{15:0}$	16.5 %	predominant	16.8 %
iso-$C_{14:0}$	12.5 %	n/d	5,7 %
anteiso-$C_{17:0}$	7.4 %	n/d	6.1 %
iso-$C_{15:0}$	7.0 %	n/d	7.8 %
$C_{18:0}$	4.8 %	n/d	trace
$C_{15:0}$	2.3 %	n/d	trace
$C_{14:0}$	2.1 %	n/d	trace
$C_{16:1}\omega7c$	1.8 %	n/d	trace
$C_{17:1}\omega7c$	0.4 %	n/d	trace
2-OH $C_{16:0}$	0.1 %	n/d	trace
Major lipid class[1]			
DPG	+	n/d	n/d
PE	+	n/d	n/d
OH-PE	+	n/d	n/d
PI	+	n/d	n/d
Isoprenoid quinone			
MK-9(H_6)	48.1%	predominant	53 %
MK-9(H_8)	26.4%	predominant	36 %
MK-9(H_4)	25.5%	minor	
References	[32]	[30]	[33]

Notes:

[1] diphosphatidylglycerol (DPG), phosphatidylethanolamine (PE), hydroxy-phosphatidylethanolamine (OH-PE), phosphatidylinositol (PI);

[2] not determined.

Table S3. Amicetin clusters of *Streptomyces vinaceusdrappus* NRRL 2363 and *Streptomyces* sp. BP-8T

Streptomyces vinaceusdrappus NRRL 2363				*Streptomyces* sp. BP-8T	
Gene	Protein ID[1]	Predicted function of gene product[2]	Length, aa	SI, %	Accession[3]
amiA	AEF16040.1	aminotransferase class IV family protein	260	78.46	WXK80757.1
amiB	AEF16041.1	Transaminase	356	87.92	WXK80758.1
amiC	AEF16042.1	NDP-hexose-chlC3	451	78.82	WXK80759.1
amiD	AEF16043.1	NDP-hexose-3-ketoreductase,	318	77.67	WXK80760.1
amiE	AEF16044.1	Cori ester adenosyl transferase	257	91.05	WXK80761.1
amiF	AEF16045.1	GNAT family N-acetyltransferase	470	99.79	WXK80762.1
amiG	AEF16046.1	Glycosyl transferring enzyme	495	99.80	WXK80763.1
amiH	AEF16047.1	Methyltransferase domain-containing protein	249	83.13	WXK80764.1
amiI	AEF16048.1	(Deoxy)cytidine deoxyribosyltransferase	182	82.84	WXK80765.1
amiJ	AEF16049.1	Cytosine gluconic acid synthetic enzyme/glycosyltransferase	403	84.86	WXK80766.1
amiK	AEF16050.1	NDP-hexose-4-ketoreductase,	316	77.39	WXK80767.1
amiL	AEF16051.1	Benzene coenzyme A synthetic enzyme	498	81.12	WXK80768.1
amiM	AEF16052.1	Para-amino benzoic acid synthetic enzyme	677	84.84	WXK80769.1
amiN	AEF16053.1	CDP-4-ketone-6-deoxyglucose-3-dehydratase	439	93.10	WXK80770.1
amiO	AEF16054.1	ABC family translocator,	559	94.45	WXK80771.1
amiP	AEF16055.1	TetR family transcriptional regulator	208	90.20	WXK80772.1
amiQ	AEF16056.1	Putative major facilitator superfamily transporter	397	90.18	WXK80773.1
amiR	AEF16057.1	Malonyl CoA-acyl carrier protein transacylase	299	78.93	WXK80774.1
amiS	AEF16058.1	Putative glycine/serine hydroxymethyltransferase	452	86.73	WXK80775.1
amiT	AEF16059.1	Nonribosomal peptide synthetase (A-PCP)	763	77.65	WXK80776.1
amiU	AEF16060.1	dTDP-Glc-4,6-dehydratase	357	90.34	WXK80777.1

[1] – Identifiers from MIBiG database (BGC0000953), originally sequences from NCBI GenBank HM748814.1.

[2] -- [5].

[3] – Accession numbers in NCBI.

Table S4. Polyketide synthases (PKSs) and nonribosomal peptide synthetases (NRPSs) in the gene clusters of *Streptomyces* sp. BP-8^{T}

Type of secondary metabolite regions	Core biosynthesis genes					As part of most similar known cluster (similarity, %)
	Locus Tag	Size, aa	GenBank Accession	*Streptomyces lydicamycinicus* TP-A0598 orthologues	*Streptomyces decoyicus* NRRL 2666^{T} orthologues	
T2PKS	ctg1_57	422	WXK81323.1	64.29%	69.23%	α naphtocyclinoic acid biosynthetic gene cluster (75%)
	ctg1_56	428	WXK81324.1	59.32%	61.03%	
T1PKS	ctg1_521	2514	WXK74739.1	35.68%	33.19%k	
NRPS	ctg1_563	1314	WXK80863.1	38.58%	85.77%	
NRPS-like	ctg1_587	1021	WXK80841.1	46.67%	40.61%	
NRPS	ctg1_596	2360	WXK81773.1	31.86%	32.52%	
NRPS	ctg1_661	803	WXK80776.1	36.94%	38.91%	Amicetin BGC (100%)
Hgl-E KS, T1PKS	ctg1_778	2332	WXK81765.1	77.92%	77.24%	
	ctg1_777	1512	WXK80662.1			
T1PKS	ctg1_1475	1438	WXK80023.1	41.47%	43.74%	
NRPS	ctg1_1476	2168	WXK80022.1	--	--	
	ctg1_1477	989	WXK80021.1	37.44%	40.83%	
	ctg1_1478	1466	WXK80020.1	45.22%	43.97%	
	ctg1_1486	1286	WXK80012.1	42.15%	34.29%	
T3PKS	ctg1_1500	352	WXK79997.1	88.89%	90.23%	
T1PKS	ctg1_1519	2602	WXK79980.1	49.64%	50.47%	
	ctg1_1520	3939	WXK81726.1	56.91%	55.20%	
	ctg1_1521	1578	WXK79979.1	73.60%	62.14%	
	ctg1_1522	1843	WXK79978.1	54.81%	52.64%	
Trans-AT PKS	ctg1_5994	527	WXK75867.1			Iso-migrastatin BGC (100%)
	ctg1_5997	3198	WXK75864.1	35.41%	44.92%	
	ctg1_5998	8077	WXK75863.1	33.57%	34.70%	
	ctg1_5999	1992	WXK75862.1	--%	--	
	ctg1_6000	777	WXK75861.1	41.10%	37.36%	
T2PKS	ctg1_6280	422	WXK75601.1	95.01%	94.30%	Spore pigment
	ctg1_6281	416	WXK75600.1	92.55%	92.55%	
T1PKS	ctg1_6546	2519	WXK75355.1	36.92%	33.62%	
T1PKS	ctg1_7220	2504	WXK74739.1	37.92%	41.02%	
T1PKS	ctg1_7541	2521	--	35.56%	36.66%	

[1] – Abbreviation: T1PKS, Type I polyketide synthase; T2PKS, Type II polyketide synthase; T3PKS, Type III polyketide synthase; NPKS, Non-ribosomal peptide synthetase; transAT-PKS, Hgl-E KS, Heterocyst glycolipid synthase-like PKS.
2 - Genes characterized by an amino acid sequence similarity below 51% are highlighted in gray.

Table S5. Antibiotic resistance genes in the genome of *Streptomyces* sp. BP-8^T

Antibiotic resistance genes	Product	Size, (aa)	GenBank Accession	Function
ctg1_54	metallo-beta-lactamase family protein	306	WXK81326.1	hydrolysis the β-lactam antibiotics
ctg1_552	ABC-efflux	644	WXK80873.1	
ctg1_6273	ABC-efflux	650	WXK75608.1	

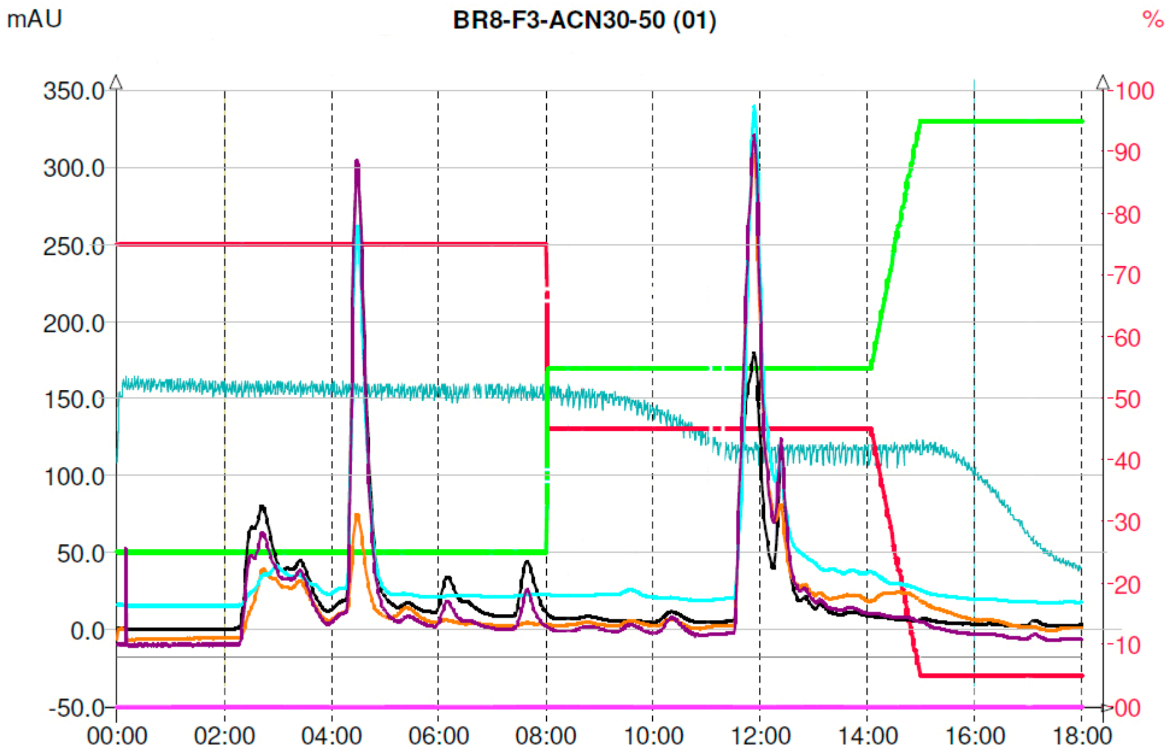

Figure S3. HPLC of the active fraction (Interchim Puriflash 4250, isocratic elution 25% of MeCN 0.1% TFA for 8 minutes, then isocratic elution 55% of MeCN 0.1% TFA for 6 minutes) using ZORBAX SB-C18 column (7 μm, 21,2×250 mm)., UV 275 nm. Active peak is at 12 min.
